# Gene-Edited Cell Models to Study Chronic Wasting Disease

**DOI:** 10.3390/v14030609

**Published:** 2022-03-15

**Authors:** Simrika Thapa, Cristobal Marrero Winkens, Waqas Tahir, Maria I. Arifin, Sabine Gilch, Hermann M. Schatzl

**Affiliations:** 1 Calgary Prion Research Unit, University of Calgary, Calgary, AB T2N 4Z6, Canada; sthapa@ucalgary.ca (S.T.); cristobal.marrerowin@ucalgary.ca (C.M.W.); waqas.tahir@inspection.gc.ca (W.T.); maria.arifin@ucalgary.ca (M.I.A.); sgilch@ucalgary.ca (S.G.); 2 Department of Comparative Biology & Experimental Medicine, Faculty of Veterinary Medicine, University of Calgary, Calgary, AB T2N 4Z6, Canada; 3 Hotchkiss Brain Institute (HBI), University of Calgary, Calgary, AB T2N 4N1, Canada; 4 Transmissible Spongiform Encephalopathies TSE Unit, NCAD Lethbridge Laboratory, Canadian Food Inspection Agency, Lethbridge, AB T1J 3Z4, Canada

**Keywords:** prion, prion disease, chronic wasting disease, CWD, gene-editing, gene-edited cells, cell culture models, CRISPR-Cas9, knock-in, knock-out

## Abstract

Prion diseases are fatal infectious neurodegenerative disorders affecting both humans and animals. They are caused by the misfolded isoform of the cellular prion protein (PrP^C^), PrP^Sc^, and currently no options exist to prevent or cure prion diseases. Chronic wasting disease (CWD) in deer, elk and other cervids is considered the most contagious prion disease, with extensive shedding of infectivity into the environment. Cell culture models provide a versatile platform for convenient quantification of prions, for studying the molecular and cellular biology of prions, and for performing high-throughput screening of potential therapeutic compounds. Unfortunately, only a very limited number of cell lines are available that facilitate robust and persistent propagation of CWD prions. Gene-editing using programmable nucleases (e.g., CRISPR-Cas9 (CC9)) has proven to be a valuable tool for high precision site-specific gene modification, including gene deletion, insertion, and replacement. CC9-based gene editing was used recently for replacing the PrP gene in mouse and cell culture models, as efficient prion propagation usually requires matching sequence homology between infecting prions and prion protein in the recipient host. As expected, such gene-editing proved to be useful for developing CWD models. Several transgenic mouse models were available that propagate CWD prions effectively, however, mostly fail to reproduce CWD pathogenesis as found in the cervid host, including CWD prion shedding. This is different for the few currently available knock-in mouse models that seem to do so. In this review, we discuss the available in vitro and in vivo models of CWD, and the impact of gene-editing strategies.

## 1. Introduction

Prion diseases are transmissible spongiform encephalopathies (TSEs) caused by the misfolded and pathological isoform of the cellular prion protein (PrP^C^), PrP^Sc^ [1,2,3]. These neurodegenerative diseases affect both animals and humans and are always fatal [3,4,5,6]. Examples of prion diseases are Creutzfeldt–Jakob disease (CJD) in humans, bovine spongiform encephalopathy (BSE) in cattle, scrapie in sheep and goats, transmissible mink encephalopathy (TME) in mink, and chronic wasting disease (CWD) in cervids [7,8,9]. Moreover, a distinguishing feature of prion diseases that sets them apart from other neurodegenerative disorders is their transmissibility within and sometimes between species, including zoonotic transmission, as was the case for BSE resulting in variant CJD (vCJD) [10,11,12,13,14,15]. BSE outbreaks and the emergence of vCJD resulted in severe and prolonged health and economic crises in various countries [11,15], serving as an example for the negative impact prion diseases can have on public health and certain economies. There are no therapeutic or prophylactic measures in place for prion diseases.

Chronic wasting disease (CWD), endemic to cervid species in North America and Scandinavia, poses a serious threat to animal health [16,17,18,19,20,21,22]. CWD is responsible for cervid population declines and has an adverse economic impact on cervid hunting and tourism industries [23,24,25,26,27,28]. CWD is considered the most contagious prion disease, and the substantial shedding of CWD prion infectivity via urine, feces, and saliva into the environment significantly contributes to disease spread [29,30,31,32]. The long-term perseverance of CWD infectivity in environment reservoirs, including soil, water, and plant sources, makes disease management very complicated [33,34,35,36,37].

Whether CWD transmits naturally to other animal species or humans is a serious matter of concern and needs continued investigation in order to control the public health burden [38,39,40]. Notably, studies have shown the possibility of experimental transmission of CWD to cattle, pigs, hamsters, cats, bank voles (BV), and non-human primates [18,21,40,41,42,43,44,45,46,47,48]. Such experimental CWD transmission to different species raises the important question of whether the range of natural hosts of CWD prions can extend beyond just cervids. Of particular importance is livestock that shares pastures contaminated for a long time with CWD prions. This enables CWD prions to indirectly enter the human food chain, thus posing a risk of zoonotic transmission of CWD to humans. CWD transmission studies in transgenic (Tg) mouse models expressing PrPs from various species including ovine, bovine, and human have revealed a low or even absent ability of CWD prions to cross relevant species barriers [49,50]. However, the transmission of CWD into non-human primates via the oral route [21,40,47,48] and efficient in vitro conversion of human PrP by CWD prions [51,52] should not be neglected. It is widely accepted that the homology between host PrP and invading prion strain plays a critical role in determining prion transmission efficiency, both for intra- and inter-species transmission [53,54,55,56]. Moreover, the existence of different CWD prion strains as well as the impact of cervid *Prnp* (gene coding for PrP) polymorphisms on disease pathogenicity, susceptibility, and transmission [57,58,59,60,61,62,63] emphasize the dynamic, emerging, and complex scenario of CWD transmissibility. In addition, the long incubation period of prion disease (years to often decades) and atypical clinical disease presentations add further layers of complexity to assessing the risk of CWD cross-species transmission. It will help to dissect the molecular and cellular biology and pathogenesis of CWD and CWD strains for defining the zoonotic potential of CWD and identifying therapeutic and prophylactic targets.

## 2. Availability of Models for Studying CWD Prions

### 2.1. Cell Culture Models

Cell culture-based in vitro models represent an important tool for analyzing the molecular and cellular biology of prion infection and can be used for high-throughput screening of anti-prion compounds. Compared to in vivo models, cell culture models are fast and cost-effective. A clear limitation is that many prion strains cannot be propagated in cell lines, including human and bovine prions (reviewed in [64]). Most of the existing cell culture models are of mouse origin and propagate only mouse-adapted scrapie strains [65,66,67,68]. For CWD cell-culture models, Raymond and coworkers (2006) developed the transformed mule deer (MD) brain-derived cell line MDB^CWD^, persistently infected with CWD prions from MD and obtained after limited dilution-based single cell cloning post-infection [69]. Interestingly, further limited dilution cloning of MDB^CWD^ cells resulted in a subclone, MDB^CWD2^, which was stained positively for fibronectin and negative for microtubule-associated protein 2 and glial fibrillary acidic protein, suggesting fibroblast origin of MDB^CWD2^ [69]. Later, RK13 cells expressing cervid PrP developed by Telling and colleagues served as an in vitro system for propagation and quantification of CWD prions [70]. RK13 cells, epithelial in origin and derived from rabbit, lack detectable PrP^C^ expression [71]. After the introduction of homologous PrP, reconstituted RK13 cells supported propagation of prions from different species, including sheep, bank vole, goat, and deer/elk—either directly from natural isolates or after prion adaptation. However, human PrP–expressing RK13 cells were not permissive to mouse-adapted CJD prions [70,71,72,73,74]. Interestingly, RK13 cells stably transfected with elk PrP^C^ initially showed no sustained propagation of CWD prions. However, after subjecting them to co-transfection with an HIV-1 Gag protein expressing plasmid and limited dilution cell cloning following infection, the Elk-21^+^ subclone was obtained. Elk-21^+^ cells were able to propagate CWD prions continuously for 67 passages [70]. Inoculation of cell lysates from Elk21^+^ cells into Tg mice expressing elk PrP resulted in clinical prion disease with phenotypical and neuropathological features as expected for CWD prions, demonstrating bona fide prion propagation in these cells. [70]. Elk-21^+^ cells were cured with dextran sulfate 500 (DS-500), designated as Elk-21^−^, and used in cervid prion cell assay (CPCA) to quantify CWD prions, either natural isolates or from experimental transmission, with sensitivities similar to prion quantification in CWD transgenic mouse models. Moreover, anti-CWD efficacy of anti-PrP antibodies, obtained from CWD vaccination in transgenic mice, was tested in this cell culture system [75]. Apart from Elk-21^+^ cells, the Telling group generated RK13 cells expressing deer PrP and infected them with mouse-adapted elk CWD prions (RKD^+^) [76]. Both Elk-21^+^ and RKD^+^ cells have been utilized to perform sensitive cell-based conformation stability assays for PrP^Sc^ to characterize prion strain properties [76]. Similarly, Hyo-Jin Kim and colleagues also generated RK13 cells expressing elk PrP, RKC1-11, which propagated CWD prions for 97 passages [77].

Although RK13 cells serve as a versatile prion replication model, they are not considered ideal to study prion biology as they are non-neuronal in origin [70,71,72]. Until now, no neuronal cell line has existed that supports persistent CWD replication. The mouse neuronal neuroblastoma cell line (N2a) failed to propagate CWD prions following expression of elk PrP, possibly due to internal resistance of N2a cells to CWD prions or dominant-negative inhibition of CWD conversion exerted by endogenous mouse PrP^C^ expression [70]. Recently, our laboratory has developed both neuronal (CAD5) and non-neuronal (mouse embryonic fibroblast, MEF) in vitro models for CWD propagation. This was achieved by expressing either cervid or BV PrP^C^ (BV-PrP) in these murine cells upon knock-out of the endogenous mouse PrP (CAD5-*Prnp*^−/−^ or MEF-*Prnp*^−/−^). Such reconstituted cells overexpressed cervid PrP or BV-PrP under a non-*Prnp* promoter. Both the reconstituted BV-PrP-expressing CAD5 and MEF cells (expressing either cervid PrP or BV-PrP) were able to propagate CWD prions successfully as suggested by prion seeding activity detected in the real-time quaking-induced conversion assay (RT-QuIC) [78]. Although such reconstituted cells supported transient replication of mouse-adapted CWD prions from MD and white-tailed deer (WTD) with low efficiency, further single cell cloning will be necessary to obtain persistently-infected cell models for CWD [78]. Moreover, different *Prnp* alleles of cervids, such as 116AG and 138SN, were expressed in CAD5-*Prnp*^−/−^ cells, which could be used in the future to characterize these *Prnp* alleles for their susceptibility to CWD prion infection [78]. The PrP-KO cells CAD5-*Prnp*^−/−^ or MEF-*Prnp*^−/−^ were generated by employing CRISPR-Cas9 (CC9)-based gene-editing strategy. Future attempts will use gene editing to generate neuronal knock-in (KI) cells, which will express cervid PrP under the authentic cervid *Prnp* promoter at normal physiological level, and test their permissiveness to persistent CWD infection. A similar approach of utilizing the CC9 system to disrupt the murine *Prnp* gene to generate CAD5-KO cells was used by another group to propagate hamster prions after exogenous introduction of hamster PrP [79]. The available cell culture models of CWD are listed in Table 1.

### 2.2. Animal Models of CWD Infection

Bioassays using animal models are indispensable in prion research and considered to be the gold standard for determining prion infectivity, infectious titers, and transmission across species. Unlike most other models of neurodegenerative diseases, animal models in prion research recapitulate the disease phenotype faithfully, e.g., accumulation of infectious prions, and PrP^Sc^ deposits and spongiform degeneration are found in the brain after experimental prion infection [80]. The most widely used animal models in prion disease research are mice (wild-type (WT) and Tg), hamsters, and to a lesser extent, BV [81]. Tg mice have played a crucial role in prion research, as Tg mice expressing the PrP sequence of the prion inoculum abrogate the species barrier that usually exists for prion transmission between species [82,83,84,85].

Tg mice expressing cervid PrP have played a significant role in studying CWD pathogenesis and transmission barriers [49,86,87,88], strain typing [57,60,89], and determining the efficacy of prophylactic and therapeutic options [75,90,91,92,93]. Back in 2004, Browning and colleagues developed a Tg mouse model for CWD, Tg(CerPrP)1536^+/−^ and Tg(CerPrP)1536^+/+^, overexpressing five- and tenfold amounts of deer PrP, respectively, in the brain compared to WT mice [86]. Intracerebral inoculation of Tg(CerPrP)1536^+/−^ and Tg(CerPrP)1536^+/+^ mice with CWD-positive MD and elk brain homogenates resulted in successful transmission of CWD prions [86]. Similarly, elk PrP-expressing transgenic mice, Tg(ElkPrP), which supported CWD propagation, were developed by two groups separately [86,87]. Later, the Prusiner group also developed Tg(ElkPrP) and Tg(DeerPrP) mice, which supported successful transmission of CWD prions from MD, WTD, and elk [49]. These Tg mice expressing cervid PrP were used to investigate CWD pathogenesis and transmission after experimental inoculation [49,86,87,88] as well as to detect the CWD infectivity in different cervid tissues, secretions, and in the environment [94,95,96,97,98]. The effect of CWD strains and cervid PrP polymorphisms on CWD susceptibility and pathogenesis was studied using cervidized Tg mice expressing various PrP polymorphisms [57,59,60,63,89,99]. Additionally, cervid PrP-expressing Tg mice were used to study cross-species transmission of BSE prions to cervids [100]. Furthermore, the anti-CWD effect of compounds and the efficacy of CWD vaccination were tested in such Tg mice [75,90,91,92,93]. The majority of cervidized Tg mouse models were generated by random integration of cervid *Prnp* transgenes against a *Prnp*^−/−^ (*Prnp*-KO) background, and are, thus, referred to as random integration transgenics (RITs) [80,86,87]. In these RITs, the cervid PrP is often expressed in *Prnp*^−/−^ mice under a foreign *Prnp* promoter (usually hamster) and the cervid PrP transgene integrates randomly into the genome in unknown copy numbers, often resulting in several-fold higher PrP expression [86,87,101]. Recently, *Prnp* gene-targeted KI mice expressing either deer or elk PrP^C^ at the normal physiological level under the *Prnp* promoter have been generated [58]. Interestingly, these KI mice, unlike the CWD RIT models, recapitulated the natural CWD transmission and prion shedding, supporting CWD infection upon peripheral challenge as well as animal co-housing [58]. Apart from transgenic mice, BVs are also susceptible to CWD infection and have been used to analyze CWD pathogenesis and for strain typing [46,102].

Non-human primates as well as Tg mice expressing human PrP were used to assess the zoonotic transmission of CWD. Non-human primates, such as squirrel monkeys and cynomolgus macaques, are ideal animal models for studying zoonotic transmission of prions as they are genetically very close to humans [11]. Interestingly, squirrel monkeys were susceptible to CWD infection, after both intracranial and oral inoculation, exhibiting typical clinical signs of prion disease and PrP^Sc^ deposition in the brain [47,48,103]. However, contrasting data exist regarding CWD transmission into cynomolgus macaques, where one group reported failure of transmission and another group showed successful CWD transmission with low attack rate and mostly atypical disease presentation, and successful transfer of prion infectivity to various rodent models [18,21,40]. Using Tg mice expressing human PrP, a complete transmission barrier was found for CWD [49,50,104]. Based on these limited studies, the zoonotic potential of CWD remains inconclusive. Further investigation should be done keeping in mind the possibilities of subclinical disease, different effects of CWD strains [20], and the longer incubation period in macaques following prion inoculation [105].

### 2.3. Ex Vivo Models of CWD Propagation

The development of ex vivo models allowed relatively fast detection of prions, including low-titer prion infectivity, with partial recapitulation of prion pathogenesis and ability to test anti-prion compounds [106,107]. Differentiated neurospheres from Tg(ElkPrP)5037^+/–^ mice overexpressing elk PrP-amplified CWD prions successfully within three weeks post-infection [108]. The prion organotypic slice culture assay (POSCA) was developed by the Aguzzi group in 2008 [109]. Such organotypic slice cultures (OSC) from 9–12 day old Tg mice expressing elk PrP (Tg12), successfully replicated CWD prions from CWD-infected brain homogenates as well as recto-anal mucosa-associated lymphoid tissue (RAMALT) [106]. Interestingly, OSCs can be used to determine the anti-prion effects of therapeutic agents [106]. Indeed, OSC recapitulated a complete three-dimensional central nervous environment, and have been successfully used to analyze scrapie prion strains in situ [107,110,111,112].

### 2.4. In Vitro Prion Amplification Assays for CWD Detection

Highly sensitive and reliable detection of prion infectivity holds a significant place in prion research. In this regard, Soto and colleagues developed an in vitro (cell-free) PrP^C^-to PrP^Sc^ conversion assay called Protein Misfolding Cyclic Amplification (PMCA). PMCA enables rapid, versatile, and sensitive detection of minute quantities of PrP^Sc^ in a sample [113]. Since then, PMCA has also been used for detection of CWD prions in brain and antemortem tissue samples, including tonsil biopsy and RAMALT, as well as in bodily fluids from CWD-infected animals [114,115,116,117,118,119]. PMCA has successfully been used for early detection of CWD prions at asymptomatic stages of the disease in various antemortem biological samples. Moreover, PMCA has been used to determine CWD species barriers and the ability of CWD prions to convert PrPs from other species. Li and colleagues incubated brain homogenates of CWD-infected elk as a seed with non-infected brain homogenates from elk, reindeer, moose, caribou, human, hamster, mouse, bovine, or sheep as substrates, and subjected them to PMCA for detection of PK-resistant PrP (PrP^res^). Very surprisingly, CWD prions could convert PrP substrates from all the species tested [120]. Moreover, Barria and colleagues utilized PMCA to test whether CWD prions could convert human PrP^C^ into PrP^res^. Excitingly, deer prions converted human PrP^C^ into PrP^Sc^, however, beforehand prion adaptation was required through successive passaging of CWD prions either in PMCA or in CWD transgenic mouse models [121]. In another study, Barria and colleagues analyzed PMCA amplification of human PrP^C^ obtained from various sources, including human brain, human-PrP-expressing Tg mouse brain, and a human-PrP^C^-overexpressing cell line, and found that human PrP was converted by CWD irrespective of the source of substrate and the polymorphism at codon 129 of human PrP [51]. More surprisingly, the biochemical properties of PrP^res^ showed similarities, unlike vCJD, with MM1 type sCJD following human PrP conversion by CWD prions [51]. These studies demonstrated the usefulness of PMCA in determining the species barrier and zoonotic potential of CWD prions.

Another ultrasensitive in vitro assay, RT-QuIC, was reported for fast detection of minute amounts of prions [122,123]. RT-QuIC measures the intensity of the fluorescent dye Thioflavin T (ThT), which binds to newly formed amyloid after seeds (prions) are incubated with the recombinant PrP (rPrP) substrate [123]. RT-QuIC is used to detect CWD prion seeding activity in a variety of biological samples from animals at different stages of prion disease. Examples are feces, urine, RAMALT, nasal brushings, saliva, blood, cerebrospinal fluid, and third eyelid from CWD-infected cervids, often at early preclinical stages [124,125,126,127,128,129,130,131,132,133]. Similar to PMCA, RT-QuIC has also been employed to assess the zoonotic potential of CWD prions. In this regard, Davenport and coworkers demonstrated that CWD prions, either from cervids or after adaptation to cats, successfully seeded human rPrP, albeit less efficiently than sCJD prions [52]. Later, Race and colleagues utilized RT-QuIC for detecting prion seeding activity in the brain and spinal cord of cynomolgus macaques experimentally inoculated with CWD prions [18]. Altogether, these results show the wide range of application of in vitro prion amplification assays in analyzing CWD prions.

## 3. Gene-Editing Strategies for Genome Engineering in the Prion Field

Recent advances in highly efficient and versatile genome-editing strategies have created new opportunities for researchers to generate gene-targeted KIs as well as KO models by introducing sequence-specific modifications into the genomes of a range of cells and animals [78,79,134,135,136,137,138,139,140,141,142,143,144,145,146,147,148]. Such gene-editing tools include zinc finger nucleases (ZFNs), transcription activator-like effector nucleases (TALENs), and RNA-guided endonucleases (RGENs), such as the clustered regularly interspaced short palindromic repeat (CRISPR)-Cas9 (CC9) system [149].

Classically, gene targeting was achieved using homologous recombination with a donor DNA template, in which an exogenous donor template replaces the endogenous gene of interest [150,151]. With the conventional homologous recombination approach, several PrP-KO and KI models were generated, including a Tg KI mouse model for CWD [58,80,81,152,153,154,155,156]. While the discovery of homologous recombination greatly advanced biomedical research, the frequency of recombination remained low in mammalian cells [157]. Moreover, homologous recombination is laborious, although highly specific, in the sense that it requires extensive selection and screening of clones to identify the ones in which the homologous recombination event occurred at the targeted endogenous gene locus [152,158]. For example, to generate the first PrP-KO Tg mouse model, homologous recombination was employed to introduce the neomycin phosphotransferase (neo) gene to replace the codons 4–187 of the 254-codon open reading frame (ORF) of the *Prnp* gene in mouse embryonic stem cells (ESCs) [152]. In order to determine the correct *Prnp*-KO clone, the selection and screening of thousands of ESC colonies was required. In this study, the frequency of homologous recombination was roughly 1 in 5000 clones [152,159]. The frequency of gene integration can, however, be increased by introducing gene-editing tools, such as ZFN, TALEN, and Cas9, alongside the homologous DNA donor template. Such endonucleases have the ability to induce targeted double-strand breaks (DSBs), which trigger cellular DNA repair mechanisms including homology directed repair (HDR), thus facilitating precise site-specific genomic modifications, including gene insertions, deletions, base substitutions, and chromosomal translocations [141,142,143,157,160,161]. In addition, DSB-causing guided endonucleases can also produce KO models in the absence of a donor template by triggering non-homologous end joining (NHEJ), which in turn causes small insertions and deletions (collectively known as indels) leading to a functional KO [78,79,137,139,146,147].

### Gene-Editing Nucleases

ZFNs are customized sequence-specific nucleases in which DNA-binding zinc-finger proteins are linked to an endonuclease domain of FokI restriction endonuclease [162]. The cleavage domain of FokI mediates the dimerization of ZFN proteins and induces a DSB within a sequence flanked by the zinc-finger proteins. Zinc-finger proteins consist of DNA-binding domains which recognize the specific sequence in the genome [163]. ZFNs have been widely used for genome manipulations, both gene insertions and deletions [135,137,139,140,164]. Interestingly, ZFNs-mediated genome editing was used for site-specific integration of the factor IX gene in hemophilia B models, and in clinical trials for treating HIV/AIDS by ZFNs-based knock-out of the HIV-1 co-receptor, CCR5 [136,165]. In the prion field, ZFN-mediated gene-editing was achieved in zebrafish, for example by generating PrP-2 (homologue of mammalian PrP) knock-out models of zebrafish in order to decipher the functions of the prion protein [166].

Similar to ZFNs, TALENs are also based on DNA-binding motifs that guide attached nucleases (usually FokI) to specific sequences within the genome, and are dimerized by FokI cleavage domain [144,167]. Usually, the assembly of TALENs occurs within 12–20 base pairs (bps) of DNA which results in their enhanced specificity for gene-editing [168]. Typically, DNA-binding domain of TALEN proteins recognize a single base pair (bp) of DNA with no overlap of target sites from neighboring domains, unlike that of zinc-finger proteins, which recognizes three bps [169,170]. As a result, ZFN is the least flexible and has the most off-target effects [144,171]. Moreover, the construction of zinc-finger arrays is difficult, making it tedious to assemble a functional nuclease, which limits the use of ZFNs as an efficient gene-editing tool [149]. TALENs are a highly specific, low cytotoxic, and flexible gene-editing tool, due to their increased and precise affinity for target bases of DNA [172,173]. However, TALENs are large proteins with highly repetitive structures, making it difficult to efficiently deliver them to cells [174]. In addition, it takes more time to customize TALEN assays and to assemble TALENs as compared to CRISPR-Cas9, while this can be achieved within a few days [144]. Several reports imply the application of TALENs as a gene-editing tool in prion research. For example, TALENs have been successfully used to generate PrP-KO mouse neuroblastoma (N2a) cells by replacing *Prnp* by a LoxP-EGFP-Zeo-LoxP knock-out cassette [142]. Moreover, TALENs-based PrP gene disruption to generate functional PrP-KO models was employed in zebrafish, mouse, and immortalized bovine fibroblasts [145,175,176].

Cas9 is another DSB-causing guided endonuclease, commonly used as CRISPR-associated nuclease in the CC9 system. Unlike customized ZFNs and TALENs, whose endonuclease domain is mediated by their DNA-binding motifs, Cas9 depends on guide RNAs (gRNAs) to reach the targeted site in the genome [144]. The CC9 strategy of gene-editing was awarded the Nobel Prize in Chemistry in 2020, and we refer the interested reader to one of the many reviews discussing its inner working [177]. Briefly, the endonuclease activity of bacterial Cas9, such as from *Streptococcus pyogenes* (SpCas9), is used for targeted cleavage on the genome by CC9 [178]. Unlike restriction endonucleases commonly used in molecular biology, Cas9 does not recognize specific DNA sequences, but can instead be directed to variable loci by a single gRNA partially complementary to the desired target sequence, due to which CC9 is the most flexible gene-editing technique [179]. Moreover, there is no need for engineering or customizing proteins such as in the case of ZFNs and TALENs, which makes CC9 a particularly user-friendly and time-saving gene-editing technique [138,144,149,180]. Once at its target site, Cas9 induces a DSB which may be repaired by NHEJ. Crucially, NHEJ has long been known to cause random deletions and insertions (collectively known as indels), thereby disrupting the gene in which the DSB occurred [181,182]. CC9-based homologous recombination has been used in cultured murine and bovine cells, as well as in fertilized bovine zygotes for *Prnp* gene disruption [78,79,147,148]. Moreover, in the presence of a donor DNA template, CC9-based targeting results in HDR allowing the site-specific introduction of exogenous homologous DNA templates at the Cas9-induced cleavage site [183]. However, HDR-dependent precise gene-editing could be limited by the possibility of NHEJ following DSB, the efficiency of which can be improved in the CC9 system by introducing genetically-encoded HDR-promoting or NHEJ-inhibiting cellular factors along with the CC9 components [184,185,186,187,188]. Using CC9-based gene targeting, KI mouse ESCs expressing hamster, BV, and PrP-EGFP fusion proteins were generated following the electroporation of CC9 vectors and KI targeted construct containing sequences of different *Prnp* alleles, homology arms, and a neomycin-resistant gene for selection [143]. Similar technology was used by our group to generate transgenic KI mice expressing cervid PrP ([189], and Arifin and Gilch, personal communication). Although the use of Cas9 is superior to other gene-editing endonucleases, it is also prone to off-target mutations. Several improvement strategies have been introduced to increase Cas9 specificity, including the combined introduction of a Cas9 nickase mutant with paired guide RNAs for DSB, which occurs only after simultaneous nicking [190], controlling the doses of Cas9 and gRNAs [191], and using Cas9 variants [192,193]. The gene-editing tools used in the generation of models in prion research are listed in Table 2.

## 4. Generation of Gene-Edited Cell Models Susceptible to CWD Prion Infection

As already mentioned above, there is a need for robust neuronal cell lines expressing cervid PrP and its polymorphic variants that are capable of propagating a wide range of CWD isolates and strains. This will serve as a versatile and robust model to study molecular and cellular aspects of CWD prion infection, and complement the animal models that recapitulate CWD pathogenesis. Studies in Tg mice have shown that the sequence of the prion protein gene (*Prnp*) expressed in the host is a major determinant of susceptibility to infection with prions from a given species [82,156], yet other cellular factors may exist [195]. Based on this concept, expressing the heterologous PrP that matches the incoming PrP^Sc^ in trans allowed propagation of such prions, including CWD prions [70,71,72]. CWD propagating cells were generated by trans-complementing or reconstituting ‘susceptible’ cells lacking PrP expression, using naturally existing KO cells (RK13) or ones made by gene-targeted disruption of the endogenous PrP (CAD5-*Prnp*^−/−^). This is important, to ensure that the endogenous PrP can no longer negatively interfere with cellular prion infection. Reconstitution was done by random integration of constructs expressing cervid or BV PrP [70,78]. The reconstitution of cells with PrP^−/−^ background was achieved by trial and error methods of stable transfection and stable lentiviral transduction [70,78]. However, random integration has its own limitations, and could have resulted in insertional mutagenesis or transgene silencing by neighboring regulatory sequences [158,196]. In addition, from in vivo Tg mouse studies, we know that cervidized Tg mice created by random integration are considered imperfect models of CWD pathogenesis, although they can propagate CWD prions upon intracerebral challenge and reproduce many CWD phenotypes [58]. They usually overexpress PrP and do not fully recapitulate peripheral CWD pathogenesis, which was solved later by the development of cervidized KI mice that mimic the natural routes of CWD transmission [58]. Likewise, cervid PrP KI cell models could serve as better models of CWD propagation. To overcome the limitations of random integration of transgenes, genome engineering of potentially susceptible cell lines can be done, with the aim to replace the endogenous PrP gene by the PrP transgene of choice. Mouse cell lines with an established good susceptibility to a wider range of prions could be employed, and to expand their range of susceptibility the endogenous *Prnp* gene locus could directly be replaced in a site-specific manner by a cervid *Prnp* or a universal acceptor PrP, allowing physiologic expression under the authentic endogenous *Prnp* promoter [78,143].

The hope is that these procedures do not negatively affect their susceptibility to persistent prion propagation now for different types and strains of prions. This is similarly true for cells supposed to propagate CWD prions. A given cell line can be non-permissive to different prion strains from the same species, why should it be for prions from a different species? For example, the widely used neuroblastoma cell line N2a supports replication of mouse-adapted scrapie strains 22L and RML, but not of Me7 and 22A [67,197]. Alternatively, not all cell lines support the replication of prions from the same species, even if there is complete sequence identity between recipient PrP and invading PrP^Sc^, obviously due to factors unrelated to the PrP sequence. For instance, Bourkas and colleagues did not observe persistent infection in gene-edited N2a-*Prnp*^−/−^ cells expressing hamster PrP following exposure to hamster prions, even though the same approach was successful in CAD5 cells [79]. Similarly, Bian and colleagues could not detect PrP^Sc^ in N2a-*Prnp*^−/−^ cells expressing elk PrP following exposure to CWD isolates, while RK13-expressing elk PrP propagated CWD prions [70]. In this line, our laboratory recently described a neuronal mouse cell line supporting CWD propagation, which is gene-edited and reconstituted CAD5 cells that supported CWD prion propagation from MD and WTD [78]. Indeed, CAD5 cells serve as an excellent neuronal cell system for prion propagation as they are permissive to the replication of a wide range of prions, including mouse-adapted scrapie and hamster prions [67,79,198].

Briefly, murine PrP-expressing CAD5 (neuronal) and MEF (non-neuronal) cells were engineered to express cervid PrP or BV PrP (universal acceptor) in the absence of endogenous PrP expression using a two-step approach, which entails CC9-mediated PrP-KO followed by lentiviral reconstitution with either cervid PrP or BV PrP [78]. Since CAD5 and MEF cells are of murine origin and express mouse PrP, they are unable to propagate CWD prions when mouse PrP is in the background. Following CC9-mediated disruption of the endogenous mouse *Prnp* locus, PrP-KO cells, CAD5-*Prnp*^−/−^ and MEF-*Prnp*^−/−^, devoid of mouse PrP were generated. Later, these gene-edited PrP-KO cells were stably transduced with recombinant lentiviruses expressing either BV or cervid PrP [78]. The resultant genome-engineered cells were able to propagate CWD prions from WTD and MD [78]. In order to knock out the endogenous murine *Prnp* in CAD5 and MEF cells, we expressed SpCas9 along with two gRNAs targeting opposite strands of *Prnp* exon 3 in these cells. For CAD5 cells, lipofectamine-based transfection of plasmids encoding Cas9 and the selected gRNAs was used [78,199]. MEF, however, are less efficiently transfected, and nucleofection was chosen to introduce the required plasmids. Single cell clones were then isolated using fluorescence-activated cell sorting (FACS) based on GFP expression and expanded for analysis by genomic DNA sequencing, anti-PrP immunoblotting and immunofluorescence staining for PrP^C^. These analyses revealed the presence of clones with a complete disruption of *Prnp*, which was further confirmed when the cells were challenged with mouse-adapted 22L scrapie prions. As expected, no proteinase K (PK)-resistant PrP (PrP^res^) could be detected in 22L-challenged MEF-*Prnp*^−/−^ or CAD5-*Prnp*^−/−^ cells in immunoblot. Wild-type (WT) MEF or CAD5 cells, however, stably propagated PrP^res^ within several passages after infection with 22L prions [78]. Next, CAD5-*Prnp*^−/−^ cells were reconstituted with BV PrP (CAD5_BV), while MEF-*Prnp*^−/−^ were reconstituted with either BV (MEF_BV) or cervid PrP (MEF_Cer). The three reconstituted cell lines were then challenged with WTD and MD prions. When examined by RT-QuIC, prion seeding activity was detected in CAD5_BV, MEF_BV, and MEF_Cer cells infected with either WTD or MD prions, however, no PrP^res^ was detected in any of the six possible cell line/prion-strain combinations in immunoblot [78]. This was not unexpected, since we used a non-cloned population, whereas many widely used cell culture models with strong PrP^res^ signal in immunoblot were established by extensive subcloning for highly susceptible clones [65,66,200]. Taken together, these data demonstrate that replacement of the endogenous mouse *Prnp* with bank vole or cervid PrP rendered CAD5 and MEF cells susceptible to infection with CWD prions. This work provides a proof-of-principle of how murine cells known to propagate prions can be genetically modified to generate cell culture models for the study of CWD prions.

**Figure 1:**
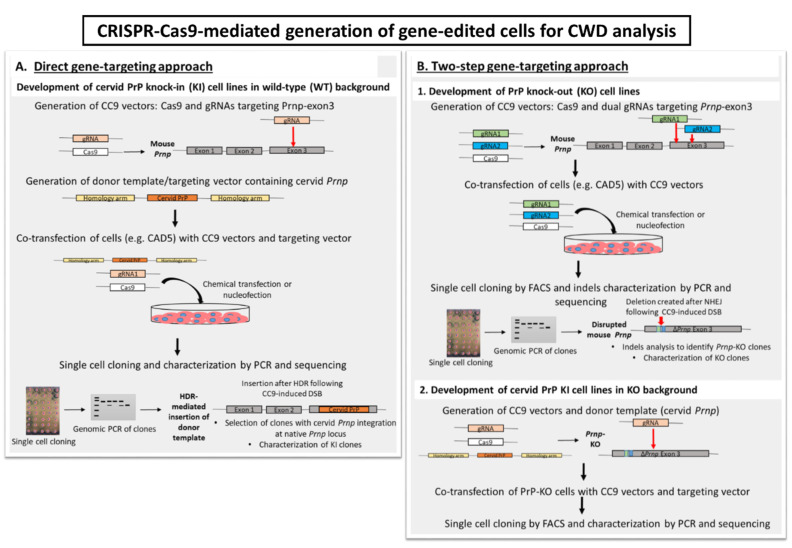
CRISPR-Cas9 (CC9)-based gene-editing for generation of cell culture models to study chronic wasting disease (CWD). Two general CC9 gene-editing approaches can be used to create CWD cell culture models: (**A**) direct gene-targeting/knock-in (KI), and (**B**) two-step gene-targeting involving knock-out of endogenous *Prnp* followed by knock-in of cervid *Prnp* at the disrupted *Prnp* locus. (**A**) Gene-targeting using CC9 in presence of a donor template (cervid *Prnp*) results in KI clones where site-specific CC9-induced double-stranded break (DSB) facilitates the homology-directed repair (HDR) mechanism by which cervid *Prnp* will replace the mouse *Prnp* in the genome. (**B**) In the two-step approach, firstly, PrP knock-out (KO) cells are generated using CC9-mediated gene disruption. By employing CC9, two DSBs can be induced in the *Prnp* gene locus of suitable mouse PrP-expressing cells, followed by gene repair by non-homologous end joining (NHEJ) resulting in indels or *Prnp* deletions. Such not-in-frame deletions or indels cause loss of functional PrP expression resulting in PrP-KO cells, as already shown for CAD5 and mouse embryonic fibroblast (MEF) *Prnp*^−/−^ models [78]. Later, PrP-KO cells can be used for gene-targeting using CC9 in presence of a cervid *Prnp* template, which will be inserted at the disrupted *Prnp* locus via HDR.

We and others used gene-editing tools to generate CAD5-*Prnp*^−/−^ cells, but PrP transgenes were introduced by random integration [78,79]. In the future, gene editing should be considered to develop KI models by homology-based integration of PrP transgenes in a site-specific manner at the host *Prnp* locus rather than through random integration. The existing PrP-ablated CAD5 and MEF KO cells could be used as a starting point for such direct gene replacement. These cells do not express PrP, so successful gene replacement events could be detected by surface PrP FACS analysis. Gene-editing strategies that could be employed to create cell culture models for CWD are shown in Figure 1.

Similar to cell culture models, gene-editing tools can be used in vivo to generate cervid PrP KI mouse models for study of CWD pathogenesis. So far, all published KI mouse models for prion research have been produced using classical homologous recombination rather than gene-editing [58,80,154,155]. Gene replacement with expression of PrP at normal physiological level under the native *Prnp* promoter was first described by Kitamoto and colleagues, who replaced the mouse *Prnp* open reading frame with human *Prnp* using a Cre-LoxP-mediated system in ESCs [201]. Another group later generated a human-*Prnp* KI mouse line that was able to propagate vCJD prions [154,155]. Since then, several gene-targeted mouse lines have been generated to express various PrPs [80,81,156]. The first KI line for CWD research, expressing WT deer and elk PrP^C^, was recently reported by Bian and colleagues [58]. These mice develop CWD and succumb to clinical disease after 200–400 dpi, depending on the inoculum-route combination [58]. Most importantly, these mice consistently propagate CWD prions administered through oral and intraperitoneal routes with similar incubation times, mimicking the natural route of CWD transmission. They perfectly recapitulate CWD pathogenesis as observed in the cervid host, with prion lateralization in the periphery and prion shedding, which is mostly absent in transgenic mouse models of CWD [58]. Although classical homologous recombination-mediated gene-targeting results in high-precision gene modifications, a major limitation is the low frequency of the desired recombination events that occurs in cells, and identifying gene-targeted clones requires extensive screening from hundreds to thousands of clones [58,152,159,202]. Using gene-editing tools like CC9 could improve the efficiency of homologous recombination [144,203]. Lately, Jackson and colleagues generated cervid-*Prnp* expressing KI ESCs using CC9, similar to their gene-edited ESCs expressing hamster PrP, BV PrP, and EGFP-PrP^C^ on a C57BL/6J background [143]. Using these ESCs, Dr. Gilch’s laboratory generated several lines of cervid-*Prnp* KI mice, expressing WT deer PrP (138SS) and the cervid polymorphic PrP variants 138NN and 116GG ([189], and Arifin and Gilch, personal communication). C57BL/6J ESCs expressing cervid PrP were injected and implanted into albino C57BL/6 mice (for ease of determining chimera percentage) and chimeric pups produced were bred and kept on a C57BL/6N background. These KI mouse lines express physiological levels of cervid PrP^C^ (in comparison to wild-type C57BL/6), and preliminary data suggested that KI mice replicate CWD prions upon peripheral challenge, reaching terminal prion disease endpoints at 400–600 days post-inoculation [189], with the typical CWD signs as seen in other CWD mouse models. Apart from these few KI lines, the majority of cervidized Tg mouse models are RITs and were generated by random integration of various cervid *Prnp* transgenes against a *Prnp*^−/−^ (*Prnp*-KO) background [80,86,87]. In these transgenics, the cervid PrP is often expressed under a foreign *Prnp* promoter (usually hamster) and the transgene integrates randomly into the genome in unknown copy numbers, often resulting in several-fold higher PrP expression [86,87,101]. The RIT models are beneficial in studying prion disease as they overexpress PrP, which leads to acceleration of disease progression and shortening of the incubation period [58,204]. However, high expression of PrP can result in the development of spontaneous neurological disease at a later stage of life [205]. Additionally, the promoter used to express PrP in RITs might result in an expression pattern in the brain different from that of normal mice [206]. During the generation of RITs, genomic positional effects can also be seen, where multiple founder lines express the transgene at different levels and in different patterns, leading sometimes to differences in phenotypes [144]. Although gene-editing CWD mouse models are still rare, they have the potential to overcome the limitations of RITs, and serve as valuable experimental tools to understand the pathobiology of CWD.

## 5. Significance and Potential Applications of Gene-Edited Cells in CWD Research

The lack of versatile cell culture models that stably propagate CWD prions limits CWD research, as in vivo studies are lengthy, expensive, and require appropriate facilities. Attempts to produce persistently prion-infected cell models often fail, and are mostly based on trial and error approaches. Traditionally, cells are derived from prion-infected animals or obtained by infecting established immortalized cell lines with prions [65,66,69,207]. In fact, the workhorses for studying prion cell biology are a very small number of murine cell lines and RK13 cells, so the majority of work is done with mouse-adapted scrapie prions. To obtain stably prion-infected cell lines and to sustain persistent infection, repeated subcloning is usually performed [64,65,67,197,200,208]. Even with subcloning, cells can lose their ability to persistently propagate prions. Interestingly, subcloning from a single clonal population can lead to either prion-susceptible or -resistant cells, just like for N2a cells where the PK1 subclone is highly susceptible, and R33-resistant to mouse-adapted scrapie prions [67]. Until now, no persistently-infected neuronal cell culture model has existed for CWD. For the generation of such neuronal models, gene-editing strategies could be advantageous. They facilitate high precision and rapid genetic alterations for *Prnp* locus-specific introduction of cervid PrP in already available murine cell lines [78]. Moreover, gene-editing strategies offer a uniform way of directly comparing the PrP substrate conversion efficiency of different cervid *Prnp* polymorphic variants, as the same locus for expression at normal physiological levels under the native promoter is targeted in a given cell line, which is impossible to achieve with a random integration approach. This uniform approach ensures minimum variability in the experimental setting. Recently, CC9-mediated base editing methods have gained popularity for creating single nucleotide polymorphism (SNPs) in the genome of cells or animals. The use of an impaired Cas nuclease and a base-modification enzyme to generate precise point mutations in the genome without inducing DSBs is gaining popularity [209,210]. Such highly efficient and precise base editing methods can be used to generate cell and mouse models expressing a range of cervid *Prnp* polymorphic variants to study the influence of polymorphism on CWD susceptibility and mechanisms involved.

Gene-edited KI cells expressing cervid PrP or a universal PrP substrate like BV PrP could provide new opportunities to study the biology of CWD prions. First, these cells could be used in scrapie cell assay (SCA) as a cellular bioassay for detection and quantification of CWD prions. SCA facilitates endpoint prion titration comparable to that achieved with animal bioassays in a rapid and cost-effective way, avoiding the need for large numbers of animals and fulfilling one of the ‘3R’ requirements in animal experimentation [211,212]. Currently, the majority of cell lines used in SCA cells express murine PrP, allowing the rapid and sensitive quantification of mouse-adapted scrapie prion titers [211,212]. Similarly, RK13 cells expressing cervid PrP are used in SCA to quantify CWD [68]. Besides quantification, cell lines used in SCA can be used for prion strain discrimination and characterization, as cells differ in their susceptibilities to different prion strains [67,211,212]. In line with this, gene-edited cell models would provide another versatile experimental platform for the study of CWD prions and newly emerging strains [57,60,89]. Second, gene-edited cells can be used in conformation-based stability assays to investigate the biochemical properties of different PrP^CWD^ to characterize prion strains [76]. Third, gene-edited CWD cell models can be utilized to study the susceptibility of cervid *Prnp* polymorphic variants to convert PrP^CWD^ in a cellular context. We already know that cervid *Prnp* polymorphisms impact CWD pathogenesis. For example, Leucine (L) homozygous at codon 132 in elk is less susceptible to CWD infection than homozygous methionine (M) [61,213]. A serine (S)/phenylalanine (F) polymorphism at codon 225 affected CWD susceptibility in MD [214]. Indeed, the finding that the ovine *Prnp* polymorphism alanine (A)_136_ arginine (R)_154_ R_171_ (ARR) is associated with resistance to scrapie infection in sheep [215] was recapitulated in RK13 cells expressing the corresponding ovine allele ARR [216]. This suggests that cell culture models are able to model susceptibility profiles of different *Prnp* alleles as observed at the animal level. Fourth, gene-edited CWD cell culture models would facilitate the rapid high-throughput screening of compounds with anti-prion efficacy, as done before for other prions [217].

## 6. Conclusions

CWD is currently the most contagious prion disease, and its zoonotic potential is yet to be determined. Moreover, for developing therapeutics against CWD it is important to understand the molecular and cellular biology of CWD strains, their intra- and inter-species transmission properties, and the influence of cervid *Prnp* polymorphism on CWD. Only very few cell culture models exist for CWD, and there is a need for developing new and improved ones. Recent advances in genome engineering provide an excellent platform to generate gene-edited cell culture and mouse models. For example, the CC9 technology could be used to generate cervid PrP-expressing KI cell and mouse models, which could better recapitulate CWD pathogenesis and support the propagation of a variety of CWD isolates. Importantly, shedding of CWD prions into the environment—a crucial hallmark of CWD prions—is not currently recapitulated in the traditional transgenic mice, but may become accessible using KI mouse models. Moreover, gene-editing could assist in introducing heterologous PrPs from different species in KI models to assess the cross-species transmission of CWD. Gene-editing also helps to investigate the impact of *Prnp* polymorphisms on CWD pathogenesis, by generating KI models expressing various cervid polymorphic alleles. Taken together, gene-edited cell and mouse models will be critical tools to better understand the biology of CWD prions.

## Figures and Tables

**Table 1:** Cell culture models of CWD infection.

Cells	Cell Type (KO Background)	Strategy to Create KO Background	Trans- Gene Expressed	Method of Transgene Inserted	Application in CWD Research	Limitations	Reference
MDB^CWD2^	Transformed MDB				a. Prion propagation: MD prions b. Anti-prion compounds testing	a. Non-neuronal fibroblast-like b. Extensive dilution cloning required c. Susceptibility to other CWD prion strains unknown	[69]
Elk-21^+^	RK13	Naturally devoid of detectable PrP	Elk PrP	Random integration following stable transfection	a. Prion propagation: elk prions [70] b. Anti-prion compounds testing [73,76] c. Cervid prion cell assay to quantify CWD prions after curing for prion infection with DS-500 [70]	a. Non-neuronal b. PrP expressed under viral promoter c. Extensive dilution cloning required d. Lack of chronic infection (infection maintained for 67 passages) e. Uninfected counterpart failed to propagate deer prions	[70]
RKC1-11	RK13	Naturally devoid of detectable PrP	Elk PrP	Random integration following lentivirus transduction	Prion propagation: CWD prions	a. Non-neuronal origin PrP expressed under viral promoter c. Extensive dilution cloning required d. Lack of chronic infection (infection maintained for 95 passages)	[77]
RKD^+^	RK13	Naturally devoid of detectable PrP	Deer PrP	Random integration following stable transfection	a. Prion propagation: mouse-adapted elk prions b. Anti-prion compounds testing	a. Non-neuronal b. PrP expressed under viral promoter	[76]
CAD5_ BV	CAD-*Prnp*^−/−^	CRISPR-CAS9	BV PrP	Random integration following lentivirus transduction	Prion propagation: mouse-adapted MD and WTD prions	a. PrP expressed under viral promoter b. Detection of infection only by using ultrasensitive RT-QuIC and not by Western blotting	[78]
MEF_BV	MEF-*Prnp*^−/−^	CRISPR-CAS9	BV PrP	Random integration following lentivirus transduction	Prion propagation: mouse-adapted MD and WTD prions	a. Non-neuronal b. PrP expressed under viral promoter c. Detection of infection only by using ultrasensitive RT-QuIC and not by Western blotting	[78]
MEF_Cer	MEF-*Prnp*^−/−^	CRISPR-CAS9	Deer PrP	Random integration following lentivirus transduction	Prion propagation: mouse-adapted MD and WTD prions	a. Non-neuronal b. PrP expressed under viral promoter c. Detection of infection only by using RT-QuIC and not Western blotting	[78]

KO: knock-out; MDB: mule deer brain cells; RK13: rabbit kidney cells; MEF: mouse embryonic fibroblast; BV: bank vole; CWD: chronic wasting disease; MD: mule deer; WTD: white-tailed deer; DS-500: dextran sulfate 500; RT-QuIC: real-time quaking-induced conversion.

**Table 2:** Gene-editing tools employed in prion research.

Gene-Editing Tools	Model Type	Gene-Edited Cells/Animal	Model Generated	Species	Advantages of the Model	Disadvantages of the Model	References
ZFNs	Animal		*prp2*-KO	Zebrafish	No adverse development phenotype observed; Gene disruption specific to *prp2* without affecting related genes; Used for understating PrP function	Cannot be directly used to study prion propagation; Possess PrP homologue, non-susceptible substrate for prion conversion	[166]
TALENs	Cellular	Murine N2a	*Prnp*-KO		Used to study protective function of PrP^C^-dependent binding of Aβ to exosomes; KI approach to insert EGFP at *Prnp* locus allowed efficient selection of PrP-KO clones	Cannot be directly used to study prion propagation	[142]
		Bovine immortalized fibroblasts	*Prnp*-KO		Moderately efficient engineering obtained with 19/66 clones with disruption in both PrP alleles; Used for somatic cell nuclear transfer to generate PrP KO embryos with no developmental defect	Cannot be directly used to study prion propagation; Non-neuronal origin;Less efficient delivery of large-sized TALEN DNA affected TALEN expression	[145]
	Animal		*prp1*-KO; dual KO of *prp1* and *prp2*	Zebrafish	No overt phenotype; Used for understating PrP function	Cannot be directly used to study prion propagation;Possess PrP homologue, non-susceptible substrate for prion conversion	[175]
		*Prnp* ^ZH3/ZH3^	*Prnp*-KO	Mouse	Lack of TALEN-induced off-target modifications and large chromosomal aberrations; Aged mice developed chronic demyelinating peripheral neuropathy reflecting crucial role of PrP in myelin maintenance	Cannot be directly used to study prion propagation	[176]
CRISPR-Cas9	Cellular	Murine N2a	*Prnp*-KO		Used to characterize molecular consequences of PrP ablation; Careful selection of CRISPR-target sites minimized off-target effects; PrP disruption achieved in N2a cells which have highly complex karyotype	Except N2a, others are non-neuronal origin; Single cell cloning was done due to lack of selection marker; Low yield of PrP disrupted clones may be due to transfection procedure	[147]
		Murine C2C12 myocytes	*Prnp*-KO				
		Mouse epithelial NMuMG	*Prnp*-KO				
		Murine CAD5	*Prnp*-KO		Eliminated dominant-negative inhibition by endogenous PrP during prion propagation following introduction of cervid and BV PrP; Neuronal cell line; Larger deletions achieved by dual-gRNAs mediated targeting of opposite strands of the *Prnp* exon 3; Reporter markers allowed efficient selection of desired clones	Cannot be directly used to study prion propagation on itself; Random integration of cervid and BV PrP in this KO could lead to positional effect as well as PrP expression is under viral promoter	[78,79]
		Mouse embryonic stem cells (ESCs)	Gene-targeted KI of hamster PrP, variants of BV-PrP, and PrP-EGFP at endogenous *Prnp* locus		KI approach helped overcoming random integration mediated positional effect; Selection markers allowed efficient desired clone isolation High CC9-mediated homologous recombination efficiency achieved	Variable performance of gRNAs required screening of multiple gRNAs	[143]
		Bovine fetal fibroblasts	*Prnp*-KO as well as EGFP-KI		Technique further used for successful *Prnp* disruption in bovine embryos	Large deletions of the targeted *PRNP* dependent on transfection conditions	[148]
		Primary fibroblasts	Myostatin (MSTN)/PrP-KO	Goat	Precise targeting achieved with efficiency of 9–70% Minimum gRNA mediated off-target effect; Simultaneous targeting of multiple genes achieved could be advantageous	Less likely to be used in prion field	[194]
	Animal	*Prnp.*Cer.WT	KI mouse line expressing wild-type cervid PrP^C^	Mouse	Physiological levels of cervid PrP^C^ expression under endogenous *Prnp* promoter in every cells and tissues; Used to study CWD propagation and effect of polymorphism on prion propagation	Longer disease incubation time when compared to random integration transgenic models	[189]
		*Prnp.*Cer.138NN	KI mouse line expressing polymorphic138NN cervid PrP^C^	Mouse			
		*Prnp.*Cer.138SN	KI mouse line expressing polymorphic138SN cervid PrP^C^	Mouse			

ZFNs: zinc finger nucleases; TALENs: transcription activator-like effector nucleases; CRISPR: clustered regularly interspaced short palindromic repeat; KO: knock out; KI: knock in; N2a: neuroblastoma cell line; BV: bank vole; EGFP: enhanced green fluorescent protein; Aβ: amyloid β.

## Data Availability

Not applicable.
